# Mutant p53 gain of function induces HER2 over-expression in cancer cells

**DOI:** 10.1186/s12885-018-4613-1

**Published:** 2018-07-03

**Authors:** A. A. Román-Rosales, E. García-Villa, L. A. Herrera, P. Gariglio, J. Díaz-Chávez

**Affiliations:** 1 Unidad de Investigación Biomédica en Cáncer, Instituto de Investigaciones Biomédicas, UNAM/Instituto Nacional de Cancerología, Av. San Fernando No. 22, Sección XVI, Tlalpan, 14080 Ciudad de México, Mexico; 2 Departamento de Genética y Biología Molecular, Centro de Investigación y de Estudios Avanzados (CINVESTAV-IPN), 07360 Ciudad de México, Mexico

**Keywords:** HER2, Mutant p53, Gain of function, Cancer

## Abstract

**Background:**

HER2 over-expression is related with a poor prognosis in patients with invasive breast cancer tumors. Clinical associations have reported that somatic mutations of p53 more frequently detected in cases of sporadic breast cancer of the HER2 subtypes, besides a high percentage of *HER2*-amplifying tumors carry germline mutations of p53. The mechanisms responsible for the acquisition of oncogenic functions of p53 mutant proteins (mtp53), known as Gain of Function (GOF), over HER2 expression have not been reported. The objective of this study was to evaluate a possible relationship between p53 mutants and *HER2* regulation.

**Methods:**

HER2 expression (transcription and protein), as well as HER2 protein stabilization have been evaluated after inducing or silencing of p53 mutants’ expression in cell lines. Finally, we evaluated the interaction of the p53 mutants over the HER2 receptor promoter.

**Results:**

Higher HER2 expression in cell lines harboring endogenous mtp53 compared with wt or null expression of p53 cell lines. Transfection of p53 mutants (R248Q and R273C) in cell lines increased the expression of HER2. Silencing of p53 mutants, decrease HER2 expression. The p53 mutants R248Q and R273C significantly increase the luciferase activity on the *HER2* promoter, and both mutants also promote acetylation of H3 and H4 histones binding in it.

**Conclusions:**

These findings show for the first time that p53 mutants induce over-expression of HER2 at transcriptional level of the HER2 protein. Our results could have clinical implications in breast cancer and other types of cancer where HER2 is over-expressed and used as a therapy target.

**Electronic supplementary material:**

The online version of this article (10.1186/s12885-018-4613-1) contains supplementary material, which is available to authorized users.

## Background

The p53 tumor suppressor protein, which is encoded by *TP53* gene, exerts its biological functions mainly by its transcriptional activity, although it is accepted that wild-type p53 (wtp53) has other biological activities that are transcription independent [1, 2]. Wild-type p53 levels are very low in normal cells; however, they rise rapidly in response to DNA damage, hypoxia, oxidant metabolism or oncogenic signaling, as well as in response to aging, maintaining genomic integrity and preventing tumor formation [3, 4]. *TP53* is the most frequent target for mutations in human cancers, with more than half of all tumors exhibiting mutation at this locus. Unlike other tumor-suppressor genes, which typically are mutated by deletion or truncation, *TP53* frequently undergoes missense mutations, resulting in single amino acid substitutions in the full length protein [3]. About a third of these missense mutations are located in six residues: R175, G245, R248, R249, R273, and R282, corresponding to the p53 DNA binding domain (DBD) and known as mutational hot-spots [5]. These p53 mutations can be roughly divided into two structural subgroups: DNA contact mutants (exemplified by R273 and R248 residues) directly involved in sequence-specific DNA contact; and conformational mutants (exemplified by the R175 residue), leading to a partial or complete abrogation of the conformational wtp53 DBD. The presence of these point mutations radically alters p53 function causing not only a simple loss of wild-type function, but also a dominant negative effect by binding and inhibiting wtp53 or a Gain of Function (GOF) acquiring novel activities independent of wtp53 [3]. p53 GOF mutations have been shown to result in oncogenic and a major proliferative processes such as increased tumorigenicity, anchorage independent cell growth and increased growth rate, increased metastasis and invasiveness, decreased sensitivity to chemotherapeutic drugs, disruption of the spindle checkpoint, activated topoisomerase I activity and induction of gene amplification, reviewed in [3]. Many of the GOF data come from p53-null systems where the expression levels of re-expressed mutant p53 were comparable to those observed in cancer cells. These results suggest a true patho-physiological role of the GOF of p53-mutants, which may lead to the development of a more aggressive cancer and poorer prognosis. The molecular mechanism of GOF’s phenotypes and up-regulation of gene expression by p53 mutants has not been clarified yet [6]. Among other important biomarkers implicated in several human cancers, there are the Human Epidermal growth factor Receptors (HERs), which regulate cell proliferation, angiogenesis, cell adhesion, cell motility, development and organogenesis, by activation of different downstream signaling pathways [7]. The HER family consists of four members (HER1–4) expressed in epithelial, mesenchymal, and neuronal cells, as well as in their cellular progenitors. Like all protein-tyrosine kinase receptors, the HER receptors exist as monomers on the cell surface and depend on their specific ligands for dimerization and trans-phosphorylation of their intracellular domains [8]. Although the product of the *ERBB2* gene (*HER2*) has no known direct activating ligand due to its constitutively activated state, this receptor is known as the preferred dimerization partner for the other family members [9]. HER2 over-expression or gene amplification occurs in 20–30% of breast cancers with a correlation of poor prognosis prior to the advent of anti-HER2 therapies. Anti-HER2 therapy is currently approved for breast, gastric, and gastroesophageal cancers, when this receptor is over-expressed. However, HER2 over-expression have been reported in other malignancies, such as bladder, cervix, colon, endometrium, germ cell, glioblastoma, head and neck, liver, lung, ovarian, pancreas and salivary duct [10]. Elucidating the mechanism leading to *HER2* gene up-regulation will be an important step to understand the pathogenesis of particularly aggressive subset of tumors over-expressing HER2 [11], as well as to find novel alternatives for therapy. Interestingly, Wilson et al. reported that patients carrying *TP53* mutations (mtp53) show a significantly higher probability of developing breast cancer with *HER2* gene amplification (83%) when compared to the cohort of early onset breast cancer cases (16%) [12]. A larger study that supports this association, found that patients with breast cancer harboring a germline *TP53* mutation, have significantly higher HER2 positive tumors prevalence, compared to their counterparts who lack any mutation [13]. There are also studies where both somatic *TP53* mutations and *HER2* gene amplification are correlated with elevated risk of breast cancer recurrence and elevated overall mortality compared with patients with neither or only one alteration [14]. It is important to note that the studies mentioned above are just clinical associations and do not propose any mechanism responsible for this relation. In this work we found, for the first time, that p53 mutants can induce up-regulation of *HER2* gene and favor the stabilization of the protein. Understanding the specific mechanisms for this relationship might reveal diagnostic and therapeutic insights in HER2 positive cancers.

## Methods

### Cell culture

Cancer cell lines: Saos-2, MCF-7, HeLa, C33A, OVCAR-3 and SKBR-3 were originally obtained from the American Type Culture Collection (ATCC). These cell lines were cultured in DMEM supplemented medium at 37 °C in a humidified 5% CO_2_ environment and were grown at 50 to 80% confluence before the next passage or further experiments.

### Transient and stable transfections

Cells were plated (5 X 10^5^ cells) in 60 mm × 15 mm culture dishes and transfected with: empty vector, p53R248Q, p53R273C [15], and wtp53 [16] plasmids; or with SureSilencing shRNA Plasmid Kit for TP53 (SABiosciences, Qiagen). Transfections were performed with Lipofectamine 2000 reagent (Invitrogen, CA) according to the manufacturer’s instructions. Transient transfections were performed for 48 h. Stable transfections were selected for 2 weeks in a growth media containing 1200 μg/ml of G-418 (Sigma-Aldrich, St Louis MO). To keep the clones selection, cells were grown continuously in media containing 800 μg/ml of G418.

### Reverse transcription and quantitative polymerase chain reaction (RT-qPCR)

Total RNA was obtained using TRIZOL reagent (Invitrogen, CA) and cDNA was reverse transcribed from 3 μg of RNA using SuperScript II reverse transcriptase (Invitrogen, CA), following the manufacturer’s recommendations. Oligonucleotide primers were designed using the Primer Express Software and purchased from Sigma-Aldrich (St Louis MO). qPCR assays were performed by using SYBR green Master Mix (Thermo Scientific, Waltham MA) in an ABI 7300 Real Time PCR system (Applied Biosystems, California USA). The qPCR program was set as follow: 95 °C for 5 min, followed by 40 cycles at 95 °C for 30 s, 60 °C for 30 s, 72 °C for 30 s and a final elongation step of 72 °C for 7 min, with a final dissociation curve. Each reaction was carried out in triplicate and targeted genes were normalized to beta-2-microglobulin (*B2M*) gene. The threshold value (CT) for each sample was used to determine gene relative expression levels by the comparative CT method (2^-ΔΔCT^ method) [17]. Detailed description of the primers used for *HER2* and *TP53* detection can be found in the Additional file 1: Table S1.

### Western blot analysis

Equal amounts of whole-cell protein extracts were separated by size on discontinuous (8 and 10%) PAGE, and transferred to a 0.45 μm nitrocellulose membrane (Millipore, Billerica MA). After blocking, membranes were probed overnight with: HER2 monoclonal primary antibody (Cell signaling, Danvers, MA) and peroxidase-conjugated antibodies: p53-DO1 (Santa Cruz Biotechnology) and β-actin (Sigma-Aldrich, St Louis MO). Secondary rabbit antibody (Sigma-Aldrich, St Louis MO) required for HER2 was employed at 1:10000 dilution. Protein expression was detected by chemiluminiscence using Supersignal West Pico (Thermo Scientific, Waltham MA). The protein band intensity was analyzed from the Western blot images by using the Syngene software.

### Promoter luciferase reporter assays

Stable transfected Saos-2 cells (empty vector, wtp53, p53R248Q, and p53R273C) were seeded at 1 × 10^5^ cells in six-wells plates and then transiently transfected with *HER2* promoter vector, pNeuLite (Addgene Inc., Cambridge MA), using Lipofectamine 2000 reagent (Invitrogen, CA) as described above. After 24 h, cells were lysed and samples probed with Dual-Glo®Luciferase Assay System (Promega, Madison WI) essentially as described in [18]. Assays were read with the Fluoroskan Ascent FL (Labsystems, Perú), 0.5 s integration/ well. Expression induction was calculated as the relative light units obtained for the Saos-2 cells expressing mtp53/average relative light units of the Saos-2 cells expressing empty vector, normalized with renilla relative light units. Results were plotted against the log of the compound concentration, using Prism 6 Software (GraphPad, San Diego CA).

### ChIP promoter assay

Chromatin immunoprecipitation (ChIP) assay over *HER2* promoter was performed in accordance to manufacturer’s kit protocol (ChIP One Day Kit, Qiagen). Briefly, cells were fixed at room temperature and washed with PBS. To obtain the cell insoluble material, cells were gently scraped with lysis buffer, collected and pelleted by centrifugation at 4 °C. DNA–protein complexes were sonicated to produce fragmented chromatin between 200 and 600 bp in length, determined by agarose gel electrophoresis. One-tenth of the fragmented chromatin was set aside as input control, and the remaining sample was pre-cleared with protein A/G. For each ChIP, either anti-p53 (DO-1) (Santa Cruz Biotechnology), anti-Histone 3 (Upstate, Millipore Merck) or anti-Histone 4 antibodies (Upstate, Millipore Merck), or non-specific control (IgG) was added and incubated overnight at 4 °C. Immune complexes were precipitated at 4 °C with magnetic beads and DNA-protein cross-links were reversed by adding NaCl. Samples were subsequently treated with proteinase K, and genomic DNA was recovered and purified by column affinity. Quantitation of DNA from p53, H3ac and H4ac ChIP, no antibody control, and input control was done with RT-qPCR as described above. Primers were designed by Primer Express Software (Applied Biosystems, Foster City, CA) and sequences are shown in Additional file 2: Table S2. Comparisons were normalized to input controls.

### Statistical analysis

Each experiment was performed at least three times, the results were presented as mean ± Standard Error of the Mean (SEM). Statistical analysis was performed with 1-tailed paired Student’s t test for means between controls and experimental data. Histograms and statistical analysis were carried out using Prism (Graphpad). * *p* < 0.05, ns = non-significant statistical difference analysis.

## Results

### HER2 and p53 expression levels in cancer cell lines

**Fig. 1:**
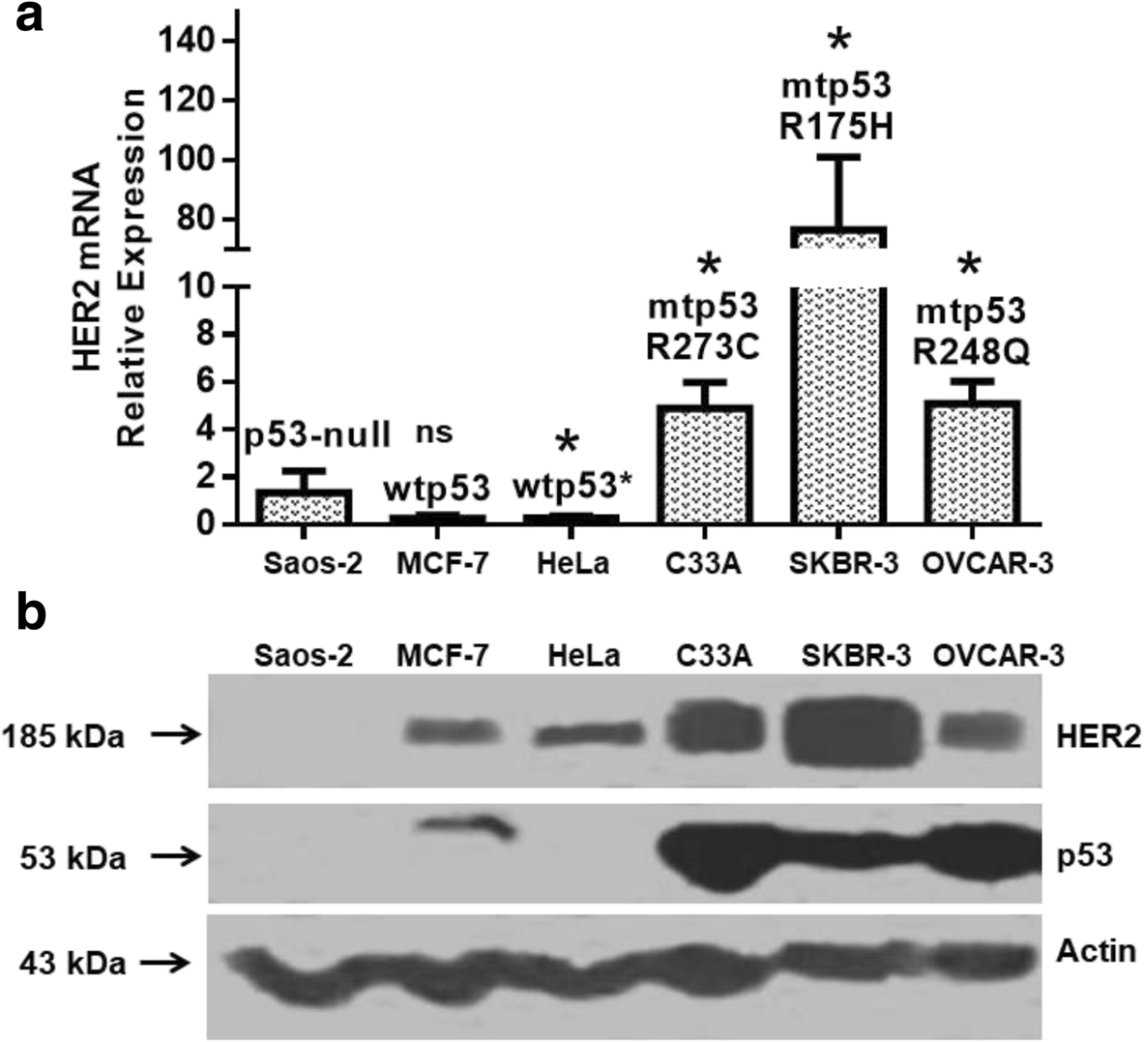
Cancer cell lines harboring p53 mutant proteins show elevated expression of both HER2 mRNA and protein. A panel of human cancer cell lines harboring p53R273C, p53R175H and p53R248Q mutant p53 proteins (C33A, SKBR-3 and OVCAR-3, respectively), as well as cells containing wild-type p53 (wtp53) protein (MCF-7 and HeLa), and null p53 expression (Saos-2) were analyzed to determine *HER2* mRNA and protein expression. HeLa cell line expresses wtp53 but its expression is decreased by Human Papillomavirus 18 (wtp53^•^). **a** *HER2* relative mRNA expression was measured by quantitative RT-PCR (RT-qPCR); final data were calculated by the comparative threshold cycle (CT) method (2^-ΔΔCT^), employing the β2-microglobulin (β2M) transcript as normalization control. The results are shown as the mean ± the standard error of the mean (SEM) of three independent assays. Statistical analysis was performed by comparing each cell line against Saos-2 cell line with t-student test, *p < 0.05; ns = non-significant statistical analysis. **b** HER2 protein levels were measured by Western Blot assay. Equal amounts of total cell lysates were blotted and proteins were identified as described in the Materials and Methods, with specific antibodies against HER2 and p53. Blots were stripped and re-probed for anti-β-actin as loading control. Image is representative of three independent experiments

As a first approach to the correlation that may exist between p53 mutant proteins and the HER2 receptor expression, we evaluated the mRNA and protein levels of p53 and HER2 in a panel of human cancer cells: MCF-7 and HeLa cells harboring wild-type p53 (wtp53), Saos-2 with null expression of p53 (p53-null), as well as, C33A, SKBR-3 and OVCAR-3 harboring three of the most frequent p53 hot-spot mutations (p53R273C, p53R175H and p53R248Q). For *HER2* mRNA expression, quantitative PCR (RT-qPCR) assays revealed that the cell lines harboring mutant p53 (mtp53) had higher expression levels when compared with the cell lines with wild-type or null p53 expression (Fig. 1a). In agreement with the mRNA expression of *HER2*, we observed a higher HER2 protein expression in the cell lines harboring mutant p53 (Fig. 1b). In Additional file 3: Figure S1, (A) we showed that exogenous wtp53 expression in HeLa cell line, counteracting HPV-E6 effects, decrease *HER2* mRNA expression; moreover, (B) wtp53 silencing in MCF-7 cell line increase *HER2* mRNA expression; (C and D) both mRNA and protein HER2 levels are insignificantly decreased in Saos-2 cells transfected with wtp53.

### The p53R248Q and p53R273C mutants increase HER2 expression

**Fig. 2:**
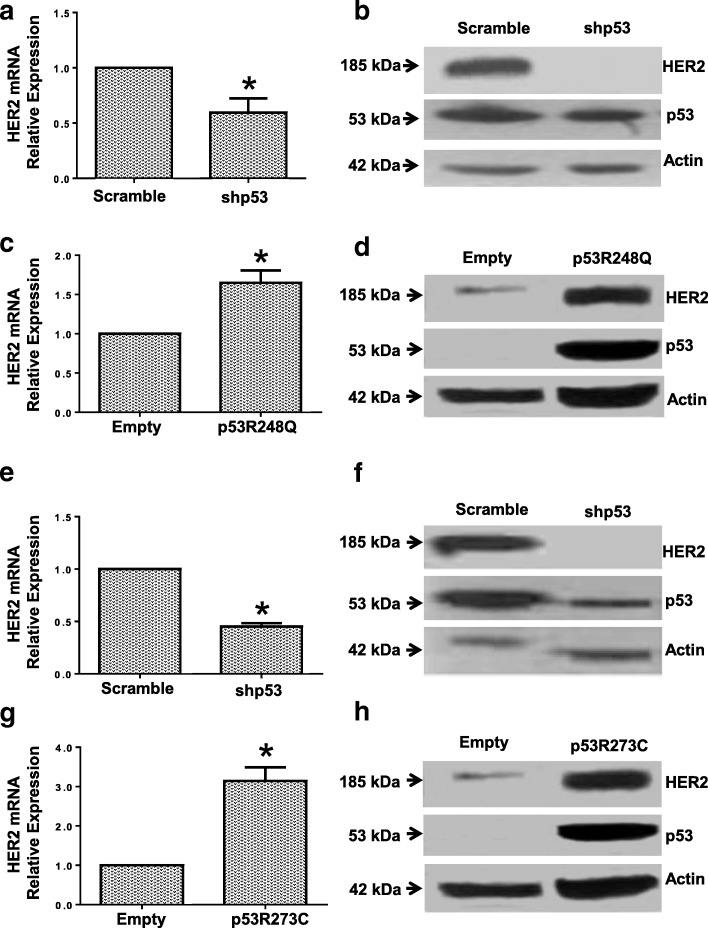
p53R248Q and p53R273C mutants positively regulates the expression of both HER2 mRNA and protein. **a**-**b** The mutant p53R248Q harbored in the OVCAR-3 cell line was inhibited by specific shRNAs for p53 (shp53), as indicated in Materials and Methods. After 48 h, transfected OVCAR-3 cells were processed to determine **a** *HER2* mRNA relative levels by RT-qPCR and **b** HER2 protein levels by Western Blot. **c**-**d** The Saos-2 cell line (p53-null) was stably transfected with mutant p53R248Q, or with the empty vector, as indicated in Materials and Methods. After stable selection, **c** *HER2* mRNA expression assay was performed by RT-qPCR, and **d** protein expression was measured by Western Blot. The RT-qPCR results are the means of at least three independent experiments ± SEM and the image of the blot is representative of three independent experiments. Statistical analysis was performed with t-student test by comparing the results obtained in: shp53 vs scramble transfected OVCAR-3 cells; or p53R248Q vs empty vector transfected Saos-2 cells: * *p* < 0.05. **e**-**f** The expression of p53R273C mutant in C33A cell line was transiently inhibited by short-hairpin RNA against p53 (shp53), under the conditions indicated in Materials and Methods. After 48 h the cells were processed for: **e** *HER2* mRNA expression assay by RT-qPCR, and **f** protein expression assay by Western Blot. **g**-**h** The Saos-2 cell line (null-p53) was stably transfected with mutant p53R273C, or with empty vector, as indicated in Materials and Methods. Transfected cells were processed after stable selection for: **g** *HER2* mRNA expression assay by RT-qPCR, and **h** protein expression assay by Western Blot. The RT-qPCR results are the means of at least three independent experiments ± SEM and the image of the blot is representative of three independent experiments. Statistical analysis was performed with t-student test by comparing the results obtained in: shp53 vs scramble transfected C33A cells; or p53R273C vs empty vector transfected Saos-2 cells: * *p* < 0.05

We then evaluated the effect of silencing or expressing exogenously two specific mutants (p53R248Q and p53R273C), on the expression of HER2. First, we silenced p53 expression in OVCAR-3 cell line which endogenously express p53R248Q mutant and observed a decreased expression of both the *HER2* mRNA and protein, when compared to the shRNA negative control plasmid (Fig. 2a and b). On the contrary, when we expressed exogenously the mutant p53R248Q by stably transfection in the Saos-2 cell line (p53-null) (Fig. 2c and d), we observed that the p53R248Q mutant, lead to an elevated mRNA and protein expression of HER2. We also transfected transiently p53R248Q mutant in the HeLa cell line with similar results than in Saos-2 cell line (Data not shown). Next, also based on our previous results where we observed an elevated expression of HER2 in C33A, a cervical cancer cell line harboring the p53R273C mutant; we silenced the endogenous expression of the p53R273C mutant in C33A cell line, and this silencing lead to a decreased in the expression in both mRNA and protein levels of HER2 as well as in the case of p53R248Q mutant (Fig. 2e and f). In counterpart, stably transfection in the p53-null cell line (Saos-2) (Fig. 2g and h), lead to an increased HER2 protein expression. We also transiently transfected the p53R273C mutant, in the HeLa cell line (data not shown), in this case we also found an increase of HER2 protein level between HeLa and Saos-2 cells, but in HeLa we did not observe an increase of *HER2* mRNA expression. These results suggest that the p53R248Q mutant, regulates positively HER2 expression at both transcriptional and protein level while regulation by p53R273C mutant could be cell context dependent.

### The p53R248Q and p53R273C mutants induce transcriptional activation of *HER2* promoter in Saos-2 cell line

**Fig. 3:**
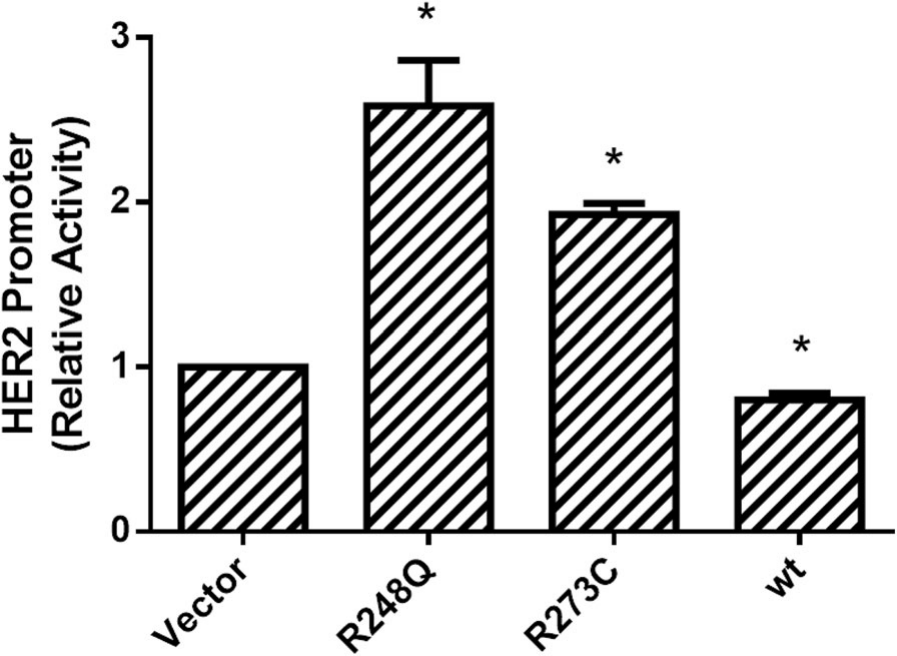
p53R248Q and p53R273C mutants but not p53 wild-type transactivate the HER2 promoter in stably transfected Saos-2 cells. Transcriptional activity of the *HER2* promoter was assessed by a Luciferase reporter assay in Saos-2 cells stably transfected with R248Q and R273C p53 mutants, as well as with wild-type p53 plasmid and empty vector. Stably transfected Saos-2 cells were co-transfected with pNeuLite luciferase reporter plasmid for the *HER2* promoter. After 48 h, luciferase activity was measured as described in the Materials and Methods. The results of the luciferase activity were normalized to renilla activity and are reported as the mean ± SEM of three independent experiments. Statistical analysis was performed with t-student test by comparing the results obtained for the Relative Activity in Saos-2 cells transfected with p53R48Q or p53R273C or wtp53 against empty vector: * *p* < 0.05

Because we also observed that p53R248Q and p53R273C mutants induce an increase in the *HER2* mRNA expression, we evaluated the effect of both mutants over the *HER2* promoter using a luciferase transactivation assay in the *HER2* promoter. We used the Saos-2 cells expressing stably either the p53R248Q or p53R273C mutants and we co-transfected the plasmid containing the HER2 promoter sequence in a luciferase reporter vector (pNeuLite) [19]. The luciferase activity was normalized with data obtained for renilla activity (Fig. 3). These results showed that, under our experimental conditions, p53R248Q and p53R273C significantly increased up to 50% the *HER2* promoter activity, with respect to the control (empty vector). We also evaluated the transient ectopic expression of wtp53 in Saos-2 cell line and in this case, the results showed that exogenous wtp53 significantly diminished the *HER2* promoter activity, in contrast to the results observed with the mutants of p53 analyzed (Fig. 3).

### The p53R248Q and p53R273C mutants bind and induce histone acetylation on the *HER2* promoter

**Fig. 4:**
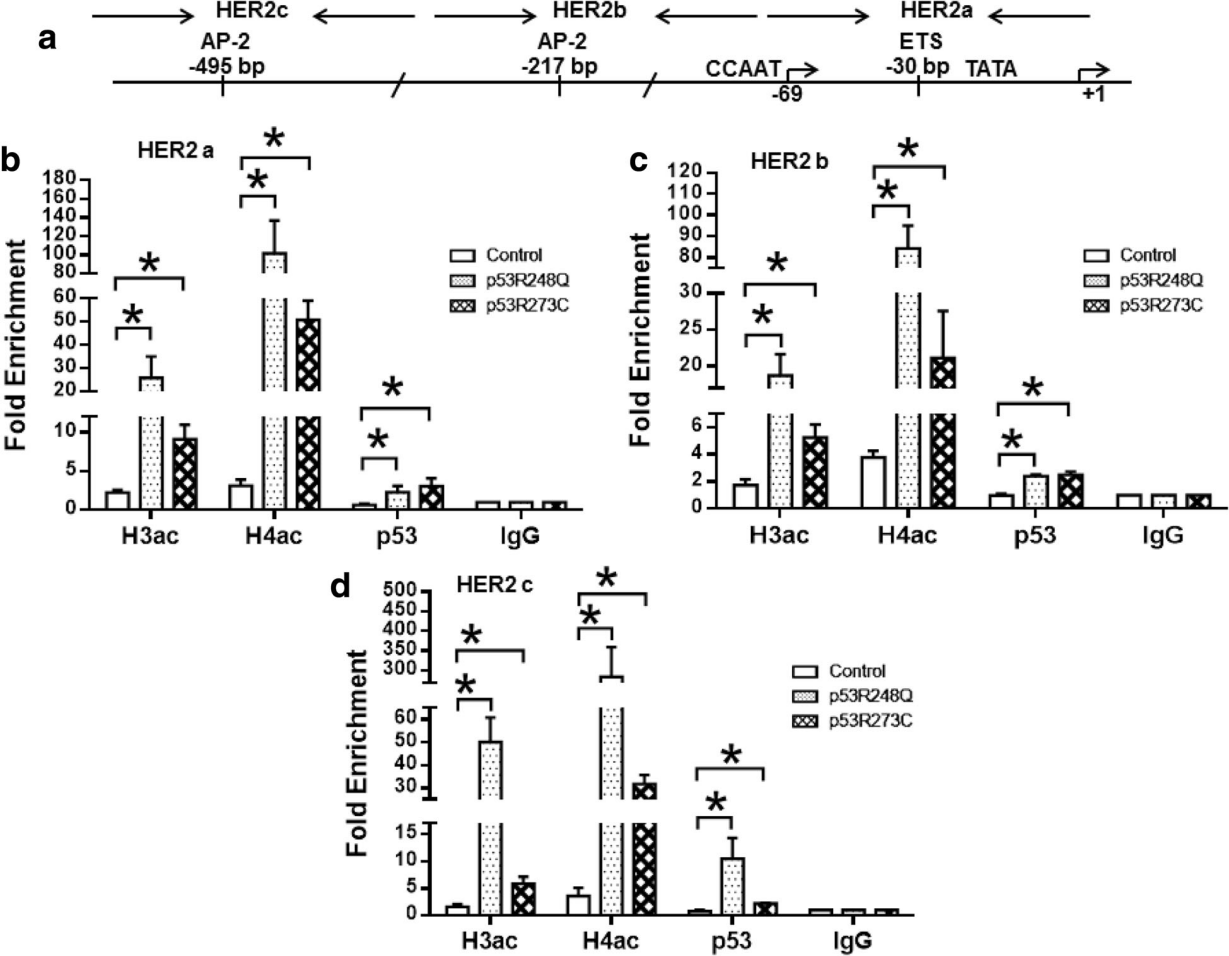
The p53R248Q and p53R273C mutants associate with chromatin and induce histone H3 and H4 acetylation in *HER2* promoter proximal region. Saos-2 cells stably transfected with p53R248Q, p53R273C or empty vector were analyzed by Chromatin immunoprecipitation (ChIP) as described in the Materials and Methods. Antibodies against acetylated histone H3, H4, p53 protein and IgG as non-relevant antibody were used for the immunoprecipitation. **a** Shows a graphic representation of the regions encompassed by the primers used for ChIP assay. Precipitated DNA was amplified with primers described in Additional file 2: Table S2. HER2a (**b**) region from − 22 to − 75 bp; HER2b (**c**) -217 bp, and (**d**) region with not known HER2 regulatory sequences. Relative amounts of acetylated H3 and H4 histones, as well as p53 interaction with HER2 promoter regions were determined on the precipitated DNA by quantitative PCR (qPCR). Normalized data were calculated relative to each primer set (**b**-**d**), comparing p52R248Q or p53R273C vs IgG ChIP 1.0% input sample. The results of Fold Enrichment are reported as the mean ± SEM of three independent experiments. Statistical analysis was performed with t-student test by comparing the results obtained for the Fold Enrichment in Saos-2 cells transfected with p53R48Q or p53R273C against empty vector transfected cells: * *p* < 0.05

**Fig. 5:**
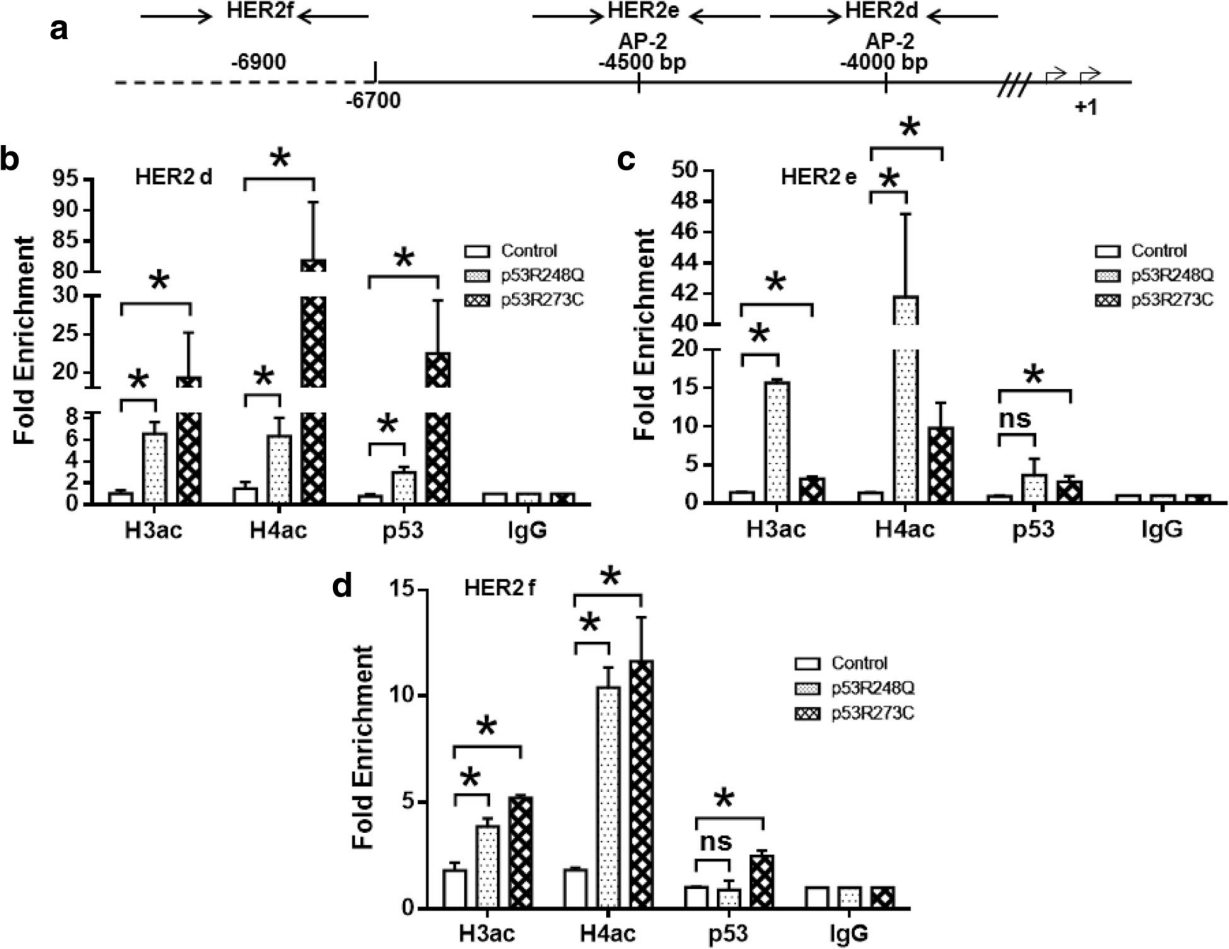
The p53R248Q and p53R273C mutants increase histone acetylation and p53 bind to *HER2* promoter distal region. Chromatin immunoprecipitation (ChIP) assays were carried out in p53R248Q, p53R273C and empty vector stably transfected Saos-2 cells as described in the Material and Methods. Antibodies against acetylated histone H3, H4, p53 protein and IgG as non-relevant antibody were used for the immunoprecipitation. **a** Shows a graphic representation of the regions encompassed by the primers used for ChIP assay. Precipitated DNA was amplified with primers described in Additional file 2: Table S2. HER2d (**b**) -4000 bp; HER2e (**c**) -4500 bp, and HER2f (**d**) up-steam the HER2 promoter. Relative amounts of acetylated H3 and H4 histones, as well as p53 interaction with HER2 distal promoter regions were determined by quantitative PCR (qPCR). Normalized data were calculated relative to each primer set (**b**-**d**), comparing p52R248Q or p53R273C vs IgG ChIP 1.0% input sample. The results of Fold Enrichment are reported as the mean ± SEM of three independent experiments. Statistical analysis was performed with t-student test by comparing the results obtained for the Fold Enrichment in Saos-2 cells transfected with p53R48Q or p53R273C against empty vector transfected cells: *p < 0.05

Due to our previous results indicating that p53 mutants up-regulate *HER2* promoter activity, we decided to gain additional insight into the molecular mechanisms underlying a possible physical interaction between the *HER2* promoter and the p53R248Q or p53R273C mutants. We performed chromatin immunoprecipitation (ChIP) assays, using antibodies directed against p53 and against acetylated forms of histones H3 and H4. These assays were performed with chromatin extracted from the Saos-2 cells stably expressing p53R248Q or p53R273C, as well as with the empty vector (control). We designed specific primers to dissect the *HER2* proximal promoter region into three PCR products, denominated as HER2a, HER2b and HER2c, according to the description reviewed by Hurst in 2001 and Delacroix in 2005 [20, 21]. The HER2a region has a TATA box in position − 22 to − 26 bp, a binding site for transcriptional factors of the ETS family in the − 30 bp position, and a CAAT (CCAAT) box in position ranging from − 71 to − 75 bp. The HER2b region, include a sequence corresponding to AP-2 transcriptional binding factor at the − 217 position. The HER2c region, also include an AP-2 binding site at the − 495 bp (Fig. 4a). We found that in HER2a and HER2b regions, both p53R248Q and p53R273C mutants had a similar fold enrichment after p53 immunoprecipitation: HER2a (2.3 and 3.0 fold enrichment, respectively) and HER2b (2.4 and 2.5, respectively); moreover, in both regions there is an important increase in the acetylation marks of H3 and H4 histones (Fig. 4b and c). For HER2c we found that p53R248Q mutant has the highest enrichment, while p53R273C mutant had a similar enrichment both HER2a and HER2c (10.4 and 2.2 fold enrichment, respectively); the most important enrichment in acetylated H3 and H4 marks was also observed in this region (Fig. 4d). The Saos-2 cell line transfected with p53R248Q presented both the highest p53 protein level and acetylated histones (particularly H4) in the three proximal regions. For the *HER2* distal promoter, we used the same set of primers reported by Delacroix et al., 2005 [22]. These primers were denominated HER2d and HER2e, which respectively cover two AP-2 binding sites located in the − 4000 bp and − 4500 bp of the *HER2* promoter. Another region upstream of the HER2 promoter, with no *HER2* activator binding sites, denominated as HER2f was included (Fig. 5a). We observed that HER2d has the major fold enrichment for the p53R273C mutant in the *HER2* promoter. In general, there was a modest enrichment of acetylated histone marks for HER2d and HER2e (Fig. 5b and c). The region denominated HER2f had the lowest enrichment for acetylation marks in H3 and H4 histones, coinciding with the lowest p53 recruitment in the promoter (Fig. 5d). It is worth noticing that in distal regions (HER2d and HER2e) the enrichment of acetylated H3 and H4 have differences between the two evaluated mutants; while p53R273C induced a higher increase in the enrichment of acetylated histone marks for HER2d region, the p53R248Q favor a higher increase of these marks in the HER2e region. Taken together, these results suggest that both the p53R248Q and p53R273C mutants are directly involved in *HER2* promoter activation. Moreover, both p53 mutants are contributing to the modification of the *HER2* promoter towards a more activated chromatin by enriching H3 and H4 acetylation marks.

## Discussion

**Fig. 6:**
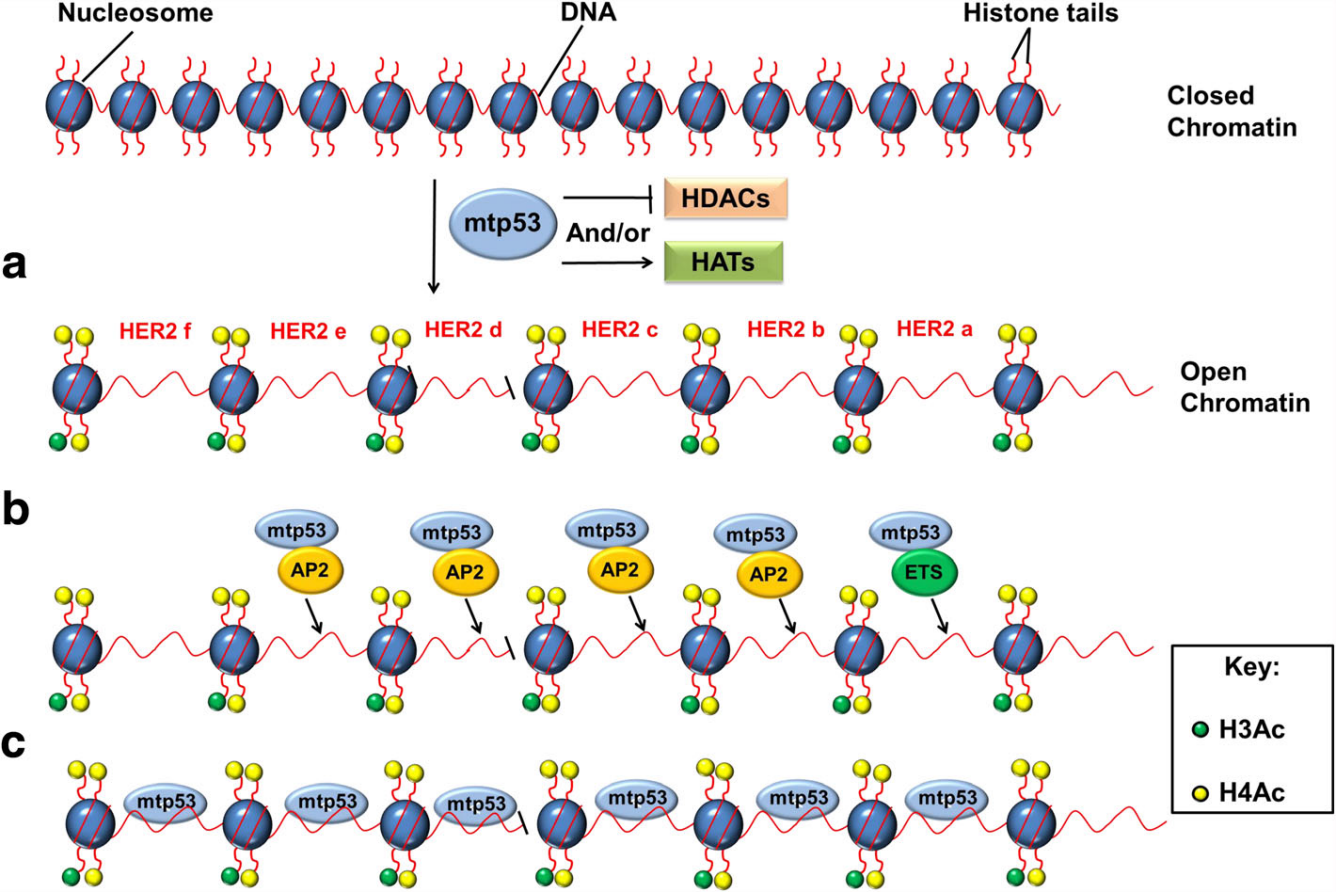
Model of HER2 up-regulation by p53 mutants. Three different molecular mechanisms of how mutant p53 may activate transcription from *HER2* promoter are depicted. **a** Mutant p53 may induce histone (s) acetylation and/or inhibit histone deacetylase (s) causing enrichment of H3 and H4 acetylated forms and chromatin reorganization on the *HER2* promoter; **b** Mutant p53 may induce interaction of one or more transcription factor (s), with the *HER2* promoter, such as AP-2 and ETS; **c** Mutant p53 itself can interact directly on the *HER2* promoter. These 3 possibilities are not mutually exclusive and would lead to the *HER2* promoter activation with the consequent *HER2* gene expression. In this representation, the histones are large blue spheres, the histone H3 acetylated marks are small green circles, the histone H4 acetylated marks are small yellow circles, the mutant p53 proteins are shown as light blue ovals, the AP-2 transcription factor are yellow ovals and ETS are green ovals. The DNA sequence is shown in red

It is generally accepted that mutant p53 (mtp53) proteins promote carcinogenesis by exerting a dominant-negative effect over wtp53 protein, as well as by new oncogenic gain-of-function (GOF) activities independent of wtp53. However, the mechanisms of the GOF phenotype by p53 mutants, are only starting to be defined [23]. In previous works, it has been observed that p53 mutants are more frequently detected in a subset of breast and gastric cancer patients with poor prognosis, such as in those presenting the HER2 subtype [24, 25]. We choose the cell lines mentioned in the Material and Methods section, because Saos-2 is a cell line known for be p53-null, which is common used to study new roles for the wild-type and mutants of p53 [26]. In the case of MCF-7 and SKBR-3 breast cancer cell lines, Xiao et al., observed that MCF-7 cells have a typical number of gene copies (i.e., 2) and very low HER2 protein content, whereas SKBR-3 cells contain approximately 10-fold more copies of the *HER2* gene and 15-fold higher HER2 protein content [27]. Consistent with Xiao’s results, we observed the most expression of mRNA and protein expression in SKBR-3 cell line. We decided not to use SKBR-3 cell line or p53R175H mutant in subsequent experiments indeed the amplification of *HER2* in this cell line [28]. In case of cervical adenocarcinomas, there are reports that conclude that amplification of *HER2* and mutations in *TP53* are rare in cervical adenocarcinomas and that, low level chromosome 17q copy number gains are not associated with HER2 over-expression [29]. Beaufort et al., described several ovarian cancer cell lines and confirms the p53 mutation for the OVCAR-3 cell line but no *HER2* amplification reported [30]. In the present study, we provided evidence to support the notion that mtp53 is implicated in the positive regulation of HER2 expression. We showed that in two different cell lines harboring wtp53 (HeLa and MCF-7), *HER2* mRNA is expressed in very low level, which agrees with the results of Yang et al. (2006) indicating that wtp53 could negatively regulate *HER2* [31]. In this regard, HeLa is a cervical cancer cell line containing the human papillomavirus-18 (HPV-18) genome that encodes for the E6 oncoprotein and causes p53 degradation [32]; which might explain the HER2 protein expression in HeLa cells. On the other hand, in spite that MCF-7 also harbors wtp53, the high basal HER2 protein levels observed in this cell line is probably inherent to the normal genetic background needed to maintain the function of breast cells; additionally, the p53 pathway has been suggested to be impaired in this tissue [33, 34]. In our work, we found that cell lines harboring three of the most common p53 mutants (p53R273C, p53R175H and p53R248Q) express higher levels of HER2 (mRNA and protein) compared to the cell lines with functional wtp53 (MCF-7), HPV-inactivated wtp53 (HeLa) or null-p53 (Saos-2). Our results are consistent with studies that suggest a clinical association between the presence of p53 mutations and HER2 over-expression [12–14]. We demonstrated that the exogenous expression of the p53R248Q or p53R273C mutants in two different cell types, HeLa and Saos-2 induces a significant increase in the levels of HER2 protein. On counterpart, we analyzed the effect of inhibiting the expression of the endogenous p53R248Q or p53R273C mutants harbored in the cell lines OVCAR-3 and C33A, respectively. We observed differences in the action efficiency of the shRNA for p53 silencing, which may be due the efficiency of transfection intrinsic to each cell line. The most p53 expression inhibition was for the p53R273C mutant expression compared with the expression for the p53R248Q mutant. Despite this, in both cases the inhibition of the p53 mutants’ expression was enough to decrease the expression of HER2, both at the level of mRNA and protein. The regulation of HER2 by p53 mutants, suggests important clinical implications in the diagnostic and therapy of HER2 positive breast cancer. To gain some insight on the molecular mechanisms by which p53 mutants positively regulate the expression of HER2, we evaluated the possibility of induction of *HER2* promoter activity. In this sense, previous reports have demonstrated the promoter activation of genes such as MDR1, EGFR, NF-kB and Axl by mtp53 activity [6, 35]. Our results showed that both p53 mutants (R248Q and R273C) stably expressed in the Saos-2 cell line induced *HER2* gene promoter activity employing a luciferase reporter assay. Up-regulation of the *HER2* promoter activity could be the result of direct mutant p53 DNA-binding, induction or recruitment of other transcription factors and transcriptional activators such as histone acetyl transferases. Among known *HER2* promoter activators are the transcription factors AP-2, YY1 and members of the ETS family [21, 36, 37]; there are also repressors such as FOXP3, PEA3 and GATA4 [19, 38]. According with the *HER2* promoter described by Hurst in 2001, we evaluated if the observed up-regulation in *HER2* is achieved through binding of mutant p53 to DNA fragments containing AP-2 or ETS sequences [21]. We demonstrated the specific recruitment of p53 mutants to both, proximal and distal regions of the promoter by ChIP assay but not all the regions were enriched at the same level. Interestingly, we observed that the highest enrichment for the p53R248Q mutant was in the *HER2* proximal region while the mutant p53R273C shows its highest recruitment level in the distal *HER2* promoter region even though both protein enrichments were located at regions with AP-2 binding sites. It is possible that the p53 mutants alone or bound with AP-2 proteins have different affinity or bind selectively to different sequences near AP-2 binding sites in the *HER2* promoter. AP-2 factors have been demonstrated to bind to wtp53 in vitro and in vivo [39, 40]; for example, in a p53-dependent binding AP-2 can regulate the *p21WAF1/CIP1* gene promoter [39, 41]. Recently it was also determined, using p53 point mutants, that the AP-2 binding region of p53 is located in amino acids 305–375 [42]. It is worth mentioning that the p53R248Q and p53R273C mutants are capable of activating AP-2 binding regions in other promoters, suggesting that these p53 mutants could cooperate with AP-2 to activate *HER2* transcription. Other transcription factors have been shown to interact with p53 mutants, including SP1, ETS1, ETS2, NF-Y, and the Vitamin D receptor [43], altering their target genes expression. Mutant p53R273H might exert its oncogenic gain-of-function by binding to ETS human genomic sequences, both within proximal and distal promoters [44]. In agreement with these studies, in the *HER2* proximal promoter there is an ETS sequence, and we observed that p53R248Q and p53R273C mutants bind to this region, suggesting that these p53 mutants interact with ETS transcription factors. On the other hand, we evaluated if these p53 mutants had any repercussion on H3 and H4 histone acetylation, enhancing the epigenetic marks and inducing directly or indirectly *HER2* promoter activity [45, 46]. As expected, histone H3 and H4 acetylation enrichment induced by p53 mutants coincides with the level of *HER2* promoter activation observed in the luciferase reporter assays. In general, the major increase in the level of the acetylated marks in H3 and particularly in H4 histones was due to p53R248Q mutant, and observed in the *HER2* proximal promoter, indicating a more relevant participation of the proximal promoter for the *HER2* activation. In agreement with our results, Zhu et al. showed that p53 GOF mutants bind to and up-regulate chromatin regulatory genes, such as the methyltransferases *MLL1*, *MLL2* and acetyltransferase *MOZ*, resulting in genome-wide increases of histone methylation and acetylation. Analysis of The Cancer Genome Atlas shows a specific tendency of up-regulation of *MLL1*, *MLL2* and *MOZ* in selected patient tumors that express GOF p53-mutants [47]. The *MOZ* gene, encodes an enzyme that adds an acetyl group to K9 of H3 histone, allowing increased gene expression [47]. In agreement with this report, we observed that p53 mutants, R248Q and R273C, increase the level of acetylated marks in H3 histone at the *HER2* promoter. In addition, we observed that the acetylation level of histone H4 was higher than that for histone H3 in all the regions analyzed, coinciding with Sandip K et al., 2001 observation, using HDAC inhibitors, suggesting that H4 acetylation is more important for *HER2* activation [46]. A schematic representation for the histone arrangement and p53 binding in the proximal and distal *HER2* promoter regions is shown in Fig. 6.

On the other hand, mutant p53 also could increase the stability of HER2 protein, previous reports had demonstrated that p53R175H can regulate some genes like EGFR at the transcriptional level, and EGFR contributes to the HER2 protein stability by increasing its dimerization state [7, 28]. In addition, it has been previously reported that HER2 protein stabilization is also induced by Hsp90 [48]. The chaperone system controls the stability of the nascent forms of both EGF-R and HER2, but only the mature form of HER2. Interestingly, it has been also reported that Hsp90 also plays a key role in the folding of p53 mutants and stabilization, suggesting that a target therapy to Hsp90 could inhibit at the same time the HER2 signaling pathway and mutant p53 gain of function [49]. Recently, it was also demonstrated that mutants of p53 promote HSF1 phosphoactivation, protein stabilization and specific DNA-binding of this protein to its target gene promoters; HSF1 is the master transcriptional regulator of heat shock chaperones, including HSP90, suggesting a mtp53-HSF1-HSP90 oncogenic cooperation to stabilize the HER2 protein [50].

## Conclusion

In conclusion, our study has demonstrated for the first time that HER2 over-expression is induced by p53 mutants through *HER2* transcriptional activation. Acetylated H3 and H4 histones are associated to the *HER2* proximal promoter in the presence of both p53R248Q and p53R273C mutants. However, more studies are required to completely elucidate the mechanism by which p53 mutants can induce *HER2* mRNA up-regulation and its protein over-expression. Exploring the oncogenic gain of functions of p53 mutants may reveal new opportunities for diagnosis and therapy. In this regard, our results could have clinical implications in breast cancer and other cancer types where HER2 is over-expressed and used as a therapy target. As we mentioned in background section, Wilson et al. reported that patients carrying TP53 mutations show a significantly higher probability of developing breast cancer with ERBB2 gene amplification in human tumors. It will be necessary to demonstrate if the p53R248Q and p53R273C mutants are associated to HER2 over-expression in cancer or if there are specific mutations of p53 associated to overexpression of HER2.

## Additional files

Additional file 1: **Table S1.** The sequences of the primers used for HER2 and p53 analysis. (PDF 339 kb)

Additional file 2: **Table S2.** The sequences of the primers used for HER2 promoter analysis. (PDF 336 kb)

Additional file 3: **Figure S1.** Wild-type p53 expression inversely correlates with *HER2* mRNA levels. HeLa (A) and Saos-2 (B) cell lines were transiently transfected with wtp53, while MCF-7 cell line (C) was transiently transfected with shp53, as indicated in Materials and Methods. Cells were processed to determine *HER2* mRNA levels by RT-qPCR. These experiments were performed by triplicate and data are shown as the mean ± SEM. Statistical analysis was performed with t-student test by comparing the results obtained in: wtp53 vs empty vector transfected HeLa and Saos-2 cells; or shp53 vs scramble transfected MCF-7 cells: * *p* < 0.05, ns = non-significant statistical difference analysis. (D) HER2 Protein expression in Saos-2 transfected with wtp53 was measured by Western Blot and the image of the blot is representative of three independent experiments. (PDF 169 kb)

## Abbreviations

ATTC: American Type Culture Collection
*B2M*: Beta-2-microglobulin
ChIP: Chromatin immunoprecipitation
CT: Threshold Cycle value
DBD: DNA Binding Domain
DMEM: Dulbecco’s Modified Eagle Medium
DNA: Deoxyribonucleic acid
EGFR: Epidermal Growth Factor Receptor
G245: Glycine in 245 codon of p53 protein
GOF: Gain of Function
HDAC: Histone Deacetylase
HER2: Epidermal Growth Factor Receptor 2
*HER2*: Epidermal Growth Factor Receptor 2 gene
mRNA: Messenger Ribonucleic acid
mtp53: Mutant p53
p53: Tumor suppressor p53
R175: Arginine in 175 codon of p53 protein
R248: Arginine in 248 codon of p53 protein
R249: Arginine in 249 codon of p53 protein
R273: Arginine in 273 codon of p53 protein
R282: Arginine in 282 codon of p53 protein
RNA: Ribonucleic acid
RT-qPCR: Reverse Transcription and quantitative Polymerase Chain Reaction
SEM: Standard Error of the Mean
shRNA: Short hairpin RNA
*TP53*: Tumor suppressor p53 gene
wtp53: Wild-type p53
