# Supplementary material for: Mutant p53 gain of function induces HER2 over-expression in cancer cells
Source: BMC Cancer. 2018 Jul 3;18:709. doi: 10.1186/s12885-018-4613-1 (PMC6029411; doi:10.1186/s12885-018-4613-1)
Supplement: Supplementary file 1 — Table S1. The sequences of the primers used for HER2 and p53 analysis. (PDF 339 kb) [file 12885_2018_4613_MOESM1_ESM.pdf]

GENE		Primer sequence	Product Size
HER2	Forward	5' – AGCTCTGCTACCAGGACACG – 3'	168 bp
	Reverse	5' – TCAGGCTCTGACAATCCTCA – 3'	
TP53	Forward	5' – GATGCTGTCCCCGGACGA – 3'	127 bp
	Reverse	5' – AGGGGCCGCCGGTGTAG – 3'	
B2M	Forward	5' – ACCCCCACTGAAAAAGATGAGT – 3'	100 bp
	Reverse	5' – ATGATGCTGCTTACATGTCTCG – 3'	
