# Supplementary material for: Mutant p53 gain of function induces HER2 over-expression in cancer cells
Source: BMC Cancer. 2018 Jul 3;18:709. doi: 10.1186/s12885-018-4613-1 (PMC6029411; doi:10.1186/s12885-018-4613-1)
Supplement: Supplementary file 2 — Table S2. The sequences of the primers used for HER2 promoter analysis. (PDF 336 kb) [file 12885_2018_4613_MOESM2_ESM.pdf]

GENE	Primer Sequence		Product Size
	Foward	Reverse	
HER2a	5’ - GTTGCCACTCCCAGACTTGT - 3’	5’ - CTCCATGGTGCTCACTGC - 3’	321 bp
HER2b	5’ - CACATCCCCCTCTTGACTA - 3’	5’ - GCAACTCCCAGCTTCACTTT - 3’	331 bp
HER2c	5’ - GGTGGCACATGCCTGTAATC - 3’	5’ - AATGGTTTTCCCACCACATC - 3’	329 bp
HER2d	5’ - ATCTGCCCTGATCTGAGTTT - 3’	5’ - GCTGCTGATTCGATTTCCTC - 3’	220 bp
HER2e	5’ - CTCAAGCCTACTCTGAGGAA - 3’	5’ - TGGAGACTGGGGGGCACAGGA -3’	302 bp
HER2f	5’ - GCAGTAGCAAGCATCGAGTT - 3’	5’ – TGGATCATCACAAAGGTTTTCA - 3’	202 bp
