# Supplementary figures and images for: Mutant p53 gain of function induces HER2 over-expression in cancer cells
Source: BMC Cancer. 2018 Jul 3;18:709. doi: 10.1186/s12885-018-4613-1 (PMC6029411; doi:10.1186/s12885-018-4613-1)

**(A)**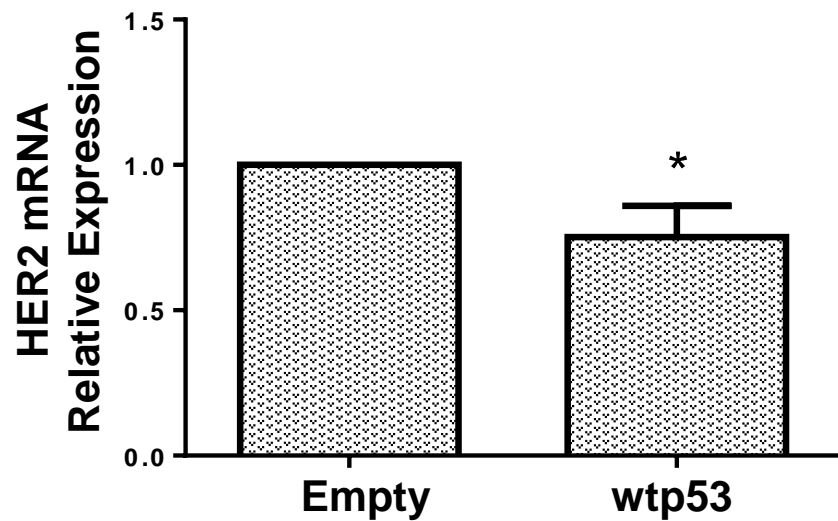**(B)**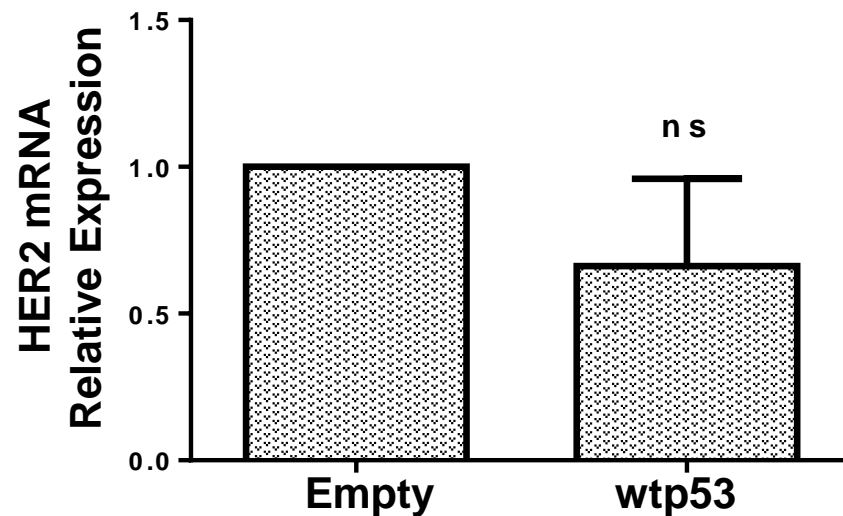**(C)**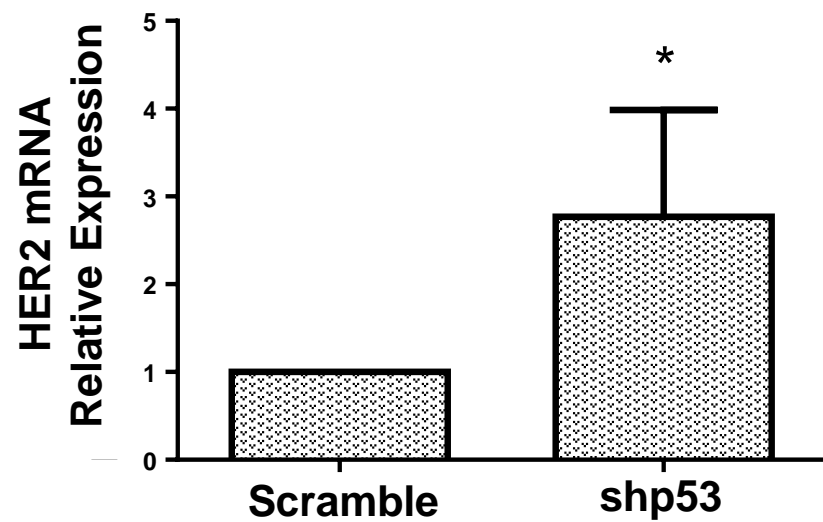**(D)**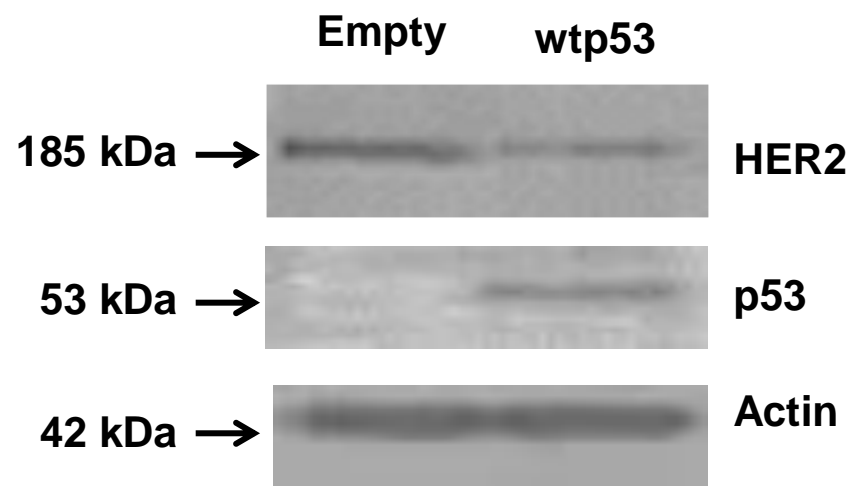

Supplement: Supplementary file 3 — Figure S1. Wild-type p53 expression inversely correlates with HER2 mRNA levels. HeLa (A) and Saos-2 (B) cell lines were transiently transfected with wtp53, while MCF-7 cell line (C) was transiently transfected with shp53, as indicated in Materials and Methods. Cells were processed to determine HER2 mRNA levels by RT-qPCR. These experiments were performed by triplicate and data are shown as the mean ± SEM. Statistical analysis was performed with t-student test by comparing the results obtained in: wtp53 vs empty vector transfected HeLa and Saos-2 cells; or shp53 vs scramble transfected MCF-7 cells: * p < 0.05, ns = non-significant statistical difference analysis. (D) HER2 Protein expression in Saos-2 transfected with wtp53 was measured by Western Blot and the image of the blot is representative of three independent experiments. (PDF 169 kb) [file 12885_2018_4613_MOESM3_ESM.pdf]
